# *In silico* identification and experimental validation of cellular uptake and intracellular labeling by a new cell penetrating peptide derived from CDN1

**DOI:** 10.1080/10717544.2021.1963352

**Published:** 2021-08-31

**Authors:** Xiangli Guo, Linlin Chen, Lidan Wang, Jingping Geng, Tao Wang, Jixiong Hu, Jason Li, Changbai Liu, Hu Wang

**Affiliations:** a Department of Pathology and Immunology, Medical School, China Three Gorges University, Yichang, China; b Hubei Key Lab of Tumor Microenvironment and Immunotherapy, China Three Gorges University, Yichang, China; c Affiliated Ren He Hospital of China Three Gorges University, Yichang, China; d The First Clinical Medical College of China Three Gorges University, Yichang, China; e College of Life Science, Yangtze University, Jingzhou, China; f Department of Biology, Johns Hopkins University, Baltimore, MD, USA; g Lead Contact

**Keywords:** Cell-permeable peptides (CPPs), bioinformatics, protein delivery, HaloTag

## Abstract

Bioactive therapeutic molecules are generally impermeable to the cell membrane, hindering their utility and efficacy. A group of peptides called cell-penetrating peptides (CPPs) were found to have the capability of transporting different types of cargo molecules across the cell membrane. Here, we identified a short peptide named P2, which has a higher proportion of basic residues than the CDN1 (cyclin-dependent kinase inhibitor 1) protein it is derived from, and we used bioinformatic analysis and experimental validation to confirm the penetration property of peptide P2. We found that peptide P2 can efficiently enter different cell lines in a concentration-dependent manner. The endocytosis pathway, especially receptor-related endocytosis, may be involved in the process of P2 penetration. Our data also showed that peptide P2 is safe in cultured cell lines and red blood cells. Lastly, peptide P2 can efficiently deliver self-labeling protein HaloTag into cells for imaging. Our study illustrates that peptide P2 is a promising imaging agent delivery vehicle for future applications.

## 1. Introduction

Many valuable bioactive molecules are impermeable to the cell membrane. Although some molecules can freely diffuse through cell barriers via certain channels and pores present in the plasma membrane, the process is extremely difficult for macromolecules (Liu et al., 2016). Therefore, developing additional pipelines that can promote macromolecules internalization is essential.

Cell-penetrating peptides (CPPs) are a class of short peptides (<30 amino acids) that can be a potential tool for the delivery of biologically active molecules. Molecules delivered include classical therapeutic peptides, nucleic acid, small molecule inhibitors, imaging probes, biomaterials, antimicrobial drugs, and anti‐cancer agents (Kardani et al., 2019). CPPs are therefore favorable delivery vehicles for the intracellular transport of various drugs to treat intractable diseases, such as cancer, organ fibrosis and neurological diseases (Liu et al., 2016; Kardani et al., 2019). Inspired by Tat (a small peptide consisting of positively charged residues from the human immunodeficiency virus type 1 trans-activator of transcription), many CPPs have been reported as biomolecular carriers. As reported in reference (Liu et al., 2016), most of the well-known CPP sequences are derived from fragments of natural protein. Traditional wet-lab experimental techniques for CPP development are costly and time-consuming (Reid et al., 2019; Wei et al., 2019). However, the rapid progress of next-generation sequencing technologies has sped up the process of protein discovery and, thus, the development of novel CPPs. Therefore, combined computational analysis and experimental validation can accelerate the discovery of new CPPs.

Generally, interactions between negatively charged cell-surface phospholipids and positively charged residues in CPPs are the initial step prior to cellular internalization. Note that charge alone is often inadequate to ensure efficient translocation (Desale et al., 2021) because other factors like charge distribution, hydrophobicity, and secondary structure are essential as well. In this study, through bioinformatics and experimental validation, we identified a novel CPP, P2, derived from the fragment of CDN1, and P2 is rich in basic residues and contains an alpha-helical structure. Through its penetration efficiency, mechanism of penetration, and *in-vitro* cytotoxicity assay results under different conditions, we confirmed that P2 is a novel CPP for cargo delivery. Furthermore, we explored intracellular labeling by using a sensitive HaloTag reporting system in cultured cell lines, and we found that P2, even at low concentration, can deliver macromolecules into the cell nucleus. Altogether, the present findings reveal that, as a novel CPP, P2 has the capability of delivering functional macromolecules efficiently.

## 2. Material and methods

### 2.1. Peptide, cell line, and cell culture

Fluorescein isothiocyanate (FITC) labeled P2 (FITC-(Acp)-RKRRQTSMTDFYHSKRRLIFSKRK) was synthesized through reversed-phase analytical high-performance liquid chromatography (>96% purity) by China Peptides (Shanghai, China). FITC-labeled control peptide NCO (FITC-(Acp)-KALGISYGRKK), TAT (FITC-(Acp)-YGRKKRRQRRR) (Wang et al., 2010, 2016; Ding et al., 2019; Zhang et al., 2019), hPP10 (FITC-(Acp)-KIPLPRFKLKCLFCKKRRKR) (Wang et al., 2016b; Geng et al., 2020), MT23 (FITC-(Acp)-LPKQKRRQRRRM) (Zhou et al., 2017) and Dot1l (FITC-(Acp)-KARKKKLNKKGRKMAGRKRGRPKK) were the same as our previous published paper. Lyophilized peptides were dissolved in phosphate-buffered saline (PBS), and then diluted to 0.5 mM in solution and stored at −20 °C until use.

Human breast cancer cell line MCF7 (P 20), human lung cancer cell line A549 (P 20), human cervical cancer cell lines HeLa (P 20), human hepatocellular carcinoma derived HepG2 (P 20), and rat hepatic stellate cell line T6 cells (P 20) were ordered from ATCC (American Type Culture Collection) and stored in our lab. All these cell lines were maintained in Dulbecco’s modified Eagle’s medium (DMEM) supplemented with 10% heated-inactivated fetal bovine serum (FBS), penicillin (100 U/ml) and streptomycin (0.1 mg/ml). All cells were grown in incubators set to 37 °C and 5% CO_2_.

### 2.2. Rat primary cultured hepatocytes

Primary hepatocytes were isolated and cultured according to the protocol previously published (Shen et al., 2012). Briefly, rat primary hepatocytes were dissociated from anesthetized adult rats by D-Hanks and two-step Pronase and Collagenase perfusion through the portal vein. After the perfusion step, the obtained rat liver tissue was cut and dissociated with a 50 ml syringe, then transferred into a 50 ml conical tube with Collagenase and incubated at 37 °C for 20 min. After filter the cell dispersion through a 200 μm pore size cell strainer into a 50 ml conical tube to remove undigested tissue fragments, the obtained hepatocytes were purified by Nycodenz density gradient. The purified hepatocytes and hepatic parenchymal cells were plated into a 24-well plate with a coverslip coated with 0.1% gelatin.

### 2.3. Prokaryotic protein expression and purification

Peptides of HaloTag were amplified through plasmids of pHaloTag-EGFP, ordered from Addgene (number #86629), and then constructed to generate pET15b-HaloTag-Dot1l, pET15b-HaloTag, and pET15b-HaloTag-P2 recombinant plasmid. HaloTag, HaloTag-P2 and HaloTag-Dot1l (Geng et al., 2020) recombinant fusion protein were expressed in the BL21 (DE3) strain of *E. coli* and induced with 0.5 mM isopropyl-β-D-thiogalactopyranoside (IPTG) at 37 °C for 16 hours, and then purified using Ni-NTA affinity chromatography (Qiagen). Protein expression and purification were monitored by SDS-PAGE. After being dialyzed with PBS and concentrated by ultrafiltration, fusion proteins were stored at −20 °C for further use.

### 2.4. Physico-chemical predictions of P2

The following physicochemical properties of P2 were predicted through the protscale tool from ExPASy (Wilkins et al., 1999): Hphob./Kyte & Doolittle, Hphob./Eisenberg et al, Accessible Residues, Average Flexibility, Bulkiness, Polarity/Grantham and Relative mutability. The hydrophobic moment of P2 was calculated using the EMBOSS server (Carver and Bleasby, 2003). The frequency of flexibility and hydrophilicity contributed by the amino acids of P2 were determined and visualized by the web-based platform of Composition Profiler (Vacic et al., 2007). The intrinsic disorder parameters of peptide P2 were also predicted by IUPred2A (Mészáros et al., 2018), PrDOS (Ishida and Kinoshita, 2007), and ANCHOR2 (Mészáros et al., 2018). A hydropathy plot was obtained from Kyte–Doolittle sliding window analysis. Conformational disorder plot (Das–Pappu plot) was conducted through the web server CIDER (Classification of Intrinsically Disordered Ensemble Regions) (Holehouse et al., 2017). The link to the webserver was listed in Table 1.

**Table 1.** List of web servers used in the text.

Webserver	Purpose	URL of webserver	Reference
ExPASy	Physico-chemical predictions	https://web.expasy.org/protscale/	(Wilkins et al., 1999)
EMBOSS	Hydrophobic moment prediction	https://www.bioinformatics.nl/cgi-bin/emboss/hmoment	(Carver & Bleasby, 2003)
Composition profiler	Flexibility and hydrophilicity prediction	http://www.cprofiler.org/	(Vacic et al., 2007)
IUPred2A, PrDOS, ANCHOR2	Disorder prediction	http://iupred2a.elte.hu http://prdos.hgc.jp/cgi-bin/top.cgi http://anchor.elte.hu/	(Mészáros et al., 2018; Ishida & Kinoshita, 2007)
CIDER	Conformational disorder prediction	http://pappulab.wustl.edu/CIDER/analysis/	(Holehouse et al., 2017)
RaptorX	Secondary structure prediction	http://raptorx.uchicago.edu/	(Wang et al., 2016)
NetSurfP	Surface accessibility, secondary structure, and disorder prediction	https://services.healthtech.dtu.dk/service.php?NetSurfP-2.0	(Klausen et al., 2019)
I-TASSER	3D structure prediction	https://zhanglab.ccmb.med.umich.edu/I-TASSER/	(Yang & Zhang, 2015)
PROCHECK, ERRAT, Verify-3D	3D structure validation	http://services.mbi.ucla.edu/SAVES/	(Colovos & Yeates, 1993; Laskowski et al., 1993; Eisenberg et al., 1997)
Ramachandran	3D structure validation	http://mordred.bioc.cam.ac.uk/_rapper/rampage.php	(Ramachandran et al., 1963)
PDB2PQR server	Electrostatic potential evaluation	https://server.poissonboltzmann.org/	(Dolinsky et al., 2004)
PPM server	Membrane interaction evaluation	https://opm.phar.umich.edu/ppm_server	(Lomize et al., 2012)
CELLPM Server	Membrane interaction evaluation	https://cellpm.org/cellpm_server	(Lomize & Pogozheva, 2018)
CPPred-FL	CPP prediction	http://server.malab.cn/CPPred-FL/	(Qiang et al., 2018)
SkipCPP-Pred	CPP prediction	http://server.malab.cn/SkipCPP-Pred/Index.html	(Wei et al., 2017a)
MLCPP	CPP prediction	http://www.thegleelab.org/MLCPP/	(Manavalan et al., 2018)
CPPred-RF	CPP prediction	http://server.malab.cn/CPPred-RF/	(Wei et al., 2017b)
CellPPD	CPP prediction	http://crdd.osdd.net/raghava/cellppd/	(Gautam et al., 2013)
Class I immunogenicity server	Immunogenicity prediction	http://tools.iedb.org/immunogenicity/	(Calis et al., 2013)
ProtLifePred server	Half-life prediction	http://protein-n-end-rule.leadhoster.com/	(Bachmair et al., 1986)

### 2.5. Structural analysis prediction of peptide P2

Based on the primary amino acid sequences of P2, its secondary structures such as α-helix, β-sheet, and backbone dynamics prediction were conducted using the RaptorX web server (Wang et al., 2016). P2’s surface accessibility, secondary structure, and disorder were also predicted through the NetSurfP webserver (Klausen et al., 2019). The three-dimensional (3D) structure of P2 was generated by I-TASSER online prediction server (Yang and Zhang, 2015). The quality of predicted models was validated with PROCHECK (Laskowski et al., 1993), ERRAT (Colovos and Yeates, 1993), Verify-3D (Eisenberg et al., 1997) patterns. Moreover, the predicted structure was also checked by the Ramachandran plot (Ramachandran et al., 1963) as well. The link to the webserver was listed in Table 1.

Input PQR files of peptide P2 were first prepared in automated mode with the PDB2PQR server (Dolinsky et al., 2004). Electrostatic surface potential maps were calculated using the adaptive Poisson–Boltzmann solver (APBS) algorithm, implemented as a plug-in in VMD or Pymol program according to default parameters. Structural analysis, molecular graphics, and geometrical property calculation were achieved with VMD or Pymol visualization program. Peptide interaction with membrane was predicted by the PPM server (Lomize et al., 2012) and CELLPM Server (Lomize and Pogozheva, 2018). The link to the webserver was listed in Table 1.

### 2.6. Penetration property prediction of peptide P2

Before the wet lab experiment of penetration property analysis, four sequence-based predictors, CPPred-FL (Qiang et al., 2018), SkipCPP-Pred (Wei et al., 2017a), MLCPP (Manavalan et al., 2018), CPPred-RF (Wei et al., 2017b) and CellPPD (Open source drug discovery consortium, 2013) were used to identify CPPs and evaluate their uptake efficiencies. Non-sense peptide NCO, well-known CPP-TAT, hPP3 (KPKRKRRKKKGHGWSR), hPP10, MT23, Scp01-b (VSRRRRRRGGRRRRGGGSYARVRRRGPRRGYARVRRRGPRR) and Dot1L from this paper’s references were also evaluated by the webserver described above. Predicted parameters including confidence or probability score were analyzed using GraphPad Prism version 7.00. The link to the webserver was listed in Table 1.

### 2.7. Cellular uptake and fluorescent microscopy

All cells (thawed cells were passaged less than 5 times) were suspended in 0.5 ml media and cultured in a 24-well plate at a concentration of 1.6 × 10^5^ cells per well overnight. After rinsing with PBS twice, the indicated cells were incubated with FITC-P2 and FITC-NCO peptides at indicated concentrations in 0.5 ml serum-free media/well for 1 hour. After incubation, the cells were washed with PBS three times and then imaged by fluorescence microscopy with 20 times lens (Nikon, Tokyo, Japan).

In the assay of cellular uptake by primary cultured cells, liver cells and hepatic parenchymal cells were placed on the coverslip and incubated for 24 hours. After rinsing with PBS twice, primary cultured cells were incubated with FITC-P2 and FITC-NCO peptides at indicated concentrations for 1 hour. Following the treatment, cells were washed with PBS and then fixed with 4% PFA. After staining with DAPI, images were captured by fluorescence microscopy (Ningbo Sunny Instruments Co., Ltd, China).

To determine the penetration efficiency of CPP, the cellular uptake of the indicated concentration of CPP was quantified by multimode spectrophotometry Peptide incubation. The washing step was the same as above, followed by cell lysate collection with 0.3 ml/well-lysing buffer (0.1 M NaOH) for 10 minutes, and then serially centrifuged at 110 g for 5 minutes. A plate-reader spectrophotometer (Tecan, Mannedorf, Switzerland) was used to quantify the fluorescence intensity of each well’s supernatant at the wavelengths of 485 nm in excitation and 535 nm in emission. The protein concentration of supernatant measured by Bradford assay was used to normalize fluorescence intensity. The fluorescence of cellular uptake was expressed as intensity per mg of total cellular protein. Experiments of quantification indicated in this paper were repeated at least three times.

### 2.8. Circular dichroism (CD) spectroscopy

Circular dichroism (CD) spectroscopy was performed on a Chirascan instrument (Applied Photophysics, Leatherhead, UK) over the range from 190 to 260 nm at 1-nm step size at 0.5 s interval. The instrument was flushed with nitrogen to remove oxygen. Three repeats were averaged for each sample. Peptide P2 (0.18 mg/ml) solution was measured in 0.1 mg/ml phosphate buffer. The spectra of the peptide P2 were recorded at 25 °C.

### 2.9. Peptide aggregation assay

To evaluate the peptide aggregation, 4 µl peptide P2 and NCO (500 µM) in PBS was run on 6.5% native polyacrylamide gel electrophoresis (PAGE) for studying peptide aggregation following the protocol published (Amit et al., 2019).

### 2.10. Cytotoxicity assay

HeLa and HepG2 cells were seeded at a density of 5000 cells per well in 96-well plates and cultured overnight. After washing with PBS, the cells were treated with different concentrations of peptide P2 for 24 hours and 48 hours. Following two times of washing, 20 µl of 5 mg/ml MTT in PBS and 80 µl of serum-free media were added into wells. After 4 hours of MTT incubation, the supernatant in the plates was removed. To dissolve the formazan crystals, 150 µl of dimethyl sulfoxide (DMSO) was added to each well and incubated at 37 °C in the dark for 15 minutes. The absorbance of DMSO-dissolved solution was read by a Multiskan Spectrum (Thermo Fisher Scientific, Waltham, MA, USA) reader at 490 nm. Experiments indicated in the paper were repeated at least three times.

### 2.11. Lactate dehydrogenase leakage assay

Cultured HeLa and MCF7 cells were seed in 96-well plates at a density of 2.5 × 10^4^ cells/per well and cultured overnight. Cells were treated with indicated concentrations of peptides for 1 hour. After peptide treatment, 20 µl cell-free supernatant was incubated with LDH reaction buffer containing substrate (50 µl each) for 5 minutes at room temperature. The absorbance of each well was read by a Multiskan Spectrum (Thermo Fisher Scientific, Waltham, MA, USA) plate reader at the wavelength of 450 nm. Experiments indicated in the paper were repeated at least three times.

### 2.12. Hemolytic activities

Mouse erythrocytes free of plasma components were isolated and purified from mouse blood by centrifugation and wash. After incubating a 20% (v/v) suspension of mouse erythrocytes in PBS with peptides at indicated concentrations, hemolytic activity was examined by the measured absorbance of the supernatant at 450 nm. A value of 100% cell lysis was measured by the incubation of erythrocytes incubated with 0.1% Triton X-100.

### 2.13. Halotag based imaging assay

The cells were grown on small, circular coverslips placed in 24-well plates and incubated for 24 hours. After washing with PBS, cells were incubated with 0.25 µg/ml or 0.4 µg/ml of prokaryotic purified HaloTag (HaloTag-P2 and HaloTag-Dot1l) for 2 hours. Following the treatment, cells were washed with serum-free media three times and then incubated with 0.25 µM of TMR (Tetramethylrhodamine) substrate for 15 minutes. Subsequently, cells were washed with PBS three times and then fixed with 4% PFA. After staining with DAPI, images were captured by Cytation 5 Cell Imaging Muti-Mode Reader (BioTek, USA).

### 2.14. Statistical analysis

All present control and experimental values are expressed as means ± standard error of the mean (SEM). Significance analysis was conducted using GraphPad software Prism 7.0 (GraphPad Software, San Diego, CA, USA). Differences of *p* < 0.05 were considered significant.

## 3. Results

### 3.1. Physicochemical properties of peptide P2

Different types of CPPs are highly diverse in their amino-acid sequences and physicochemical and biological properties. The broad CPP sequence diversity largely contributes to insufficient understanding of the structure-activity relationship of cellular uptake. It is important to note the relative abundance of positively charged residues, such as arginine or lysine (referred to as polycationic), and sequences containing an alternative pattern of charged residues, such as non-polar, hydrophobic, and polar amino acids (referred to as amphipathic) (Kardani et al., 2019). Even though most of the well-known and widely used CPPs are rich in positively charged residues, non-charged amino acids, such as leucine and tryptophan, are also crucial for CPP uptake (Schmidt et al., 2017).

As a segment of CDN1, peptide P2 is a PAR (Poly (ADP-ribose)) binding motif (Pleschke et al., 2000). P2 has been shown to contain all the types of amino acids (high proportion of basic residues) that cell penetration requires, but whether peptide P2 has translocation potential had not been previously studied.

Before we conducted wet-lab validation of peptide P2’s penetration properties, physical-chemical properties of peptide P2 were calculated. The hydrophobic property of peptide P2 was analyzed using the method of Kyte & Doolittle and Eisenberg et al’s on ExPASy Protscale. As shown in Figure S1(A), most peptide P2 residues are hydrophilic except the residues at around the middle. Highly accessible residues and average flexibility are located at both ends of the peptide (Figure S1(B)). As the mean vector sum of the hydrophobicities of the peptide side chain, the hydrophobic moment (μH) quantifies amphipathicity (Figure S1(C)). The bulkiness of side chains increases from residue 5 to residue 20 (Figure S1(D)), while the polarity (Figure S1(E)) and relative mutability (Figure S1(F)) decrease from N-terminal to C-terminal. The disorder propensity (Figure S1(G)) and flexibility (Figure S1(H)) of peptide P2 were analyzed per amino acid using the SwissProt 51 database and Composition Profiler. Peptide P2’s sequence is rich in disorder-promoting residues (Arg and Lys). Moreover, IUPred2A, ANCHOR2 and PrDOS were used to predict disorder based on the peptide basic biophysical properties (Figure S1(I)). Peptide P2 was predicted to belong to the R4 region of the Das-Pappu plot in Figure S2(A), which suggested that peptide P2 forms swollen coils in an aqueous solution. In Figure S2(B), free energies of transfer ΔG (kcal/mol) from water to n-octanol (whole residue Wimley-White hydrophobicity scale) were used to measure amino acid hydrophobicity and hydrophilicity distribution in peptide P2.

**Figure 1.**
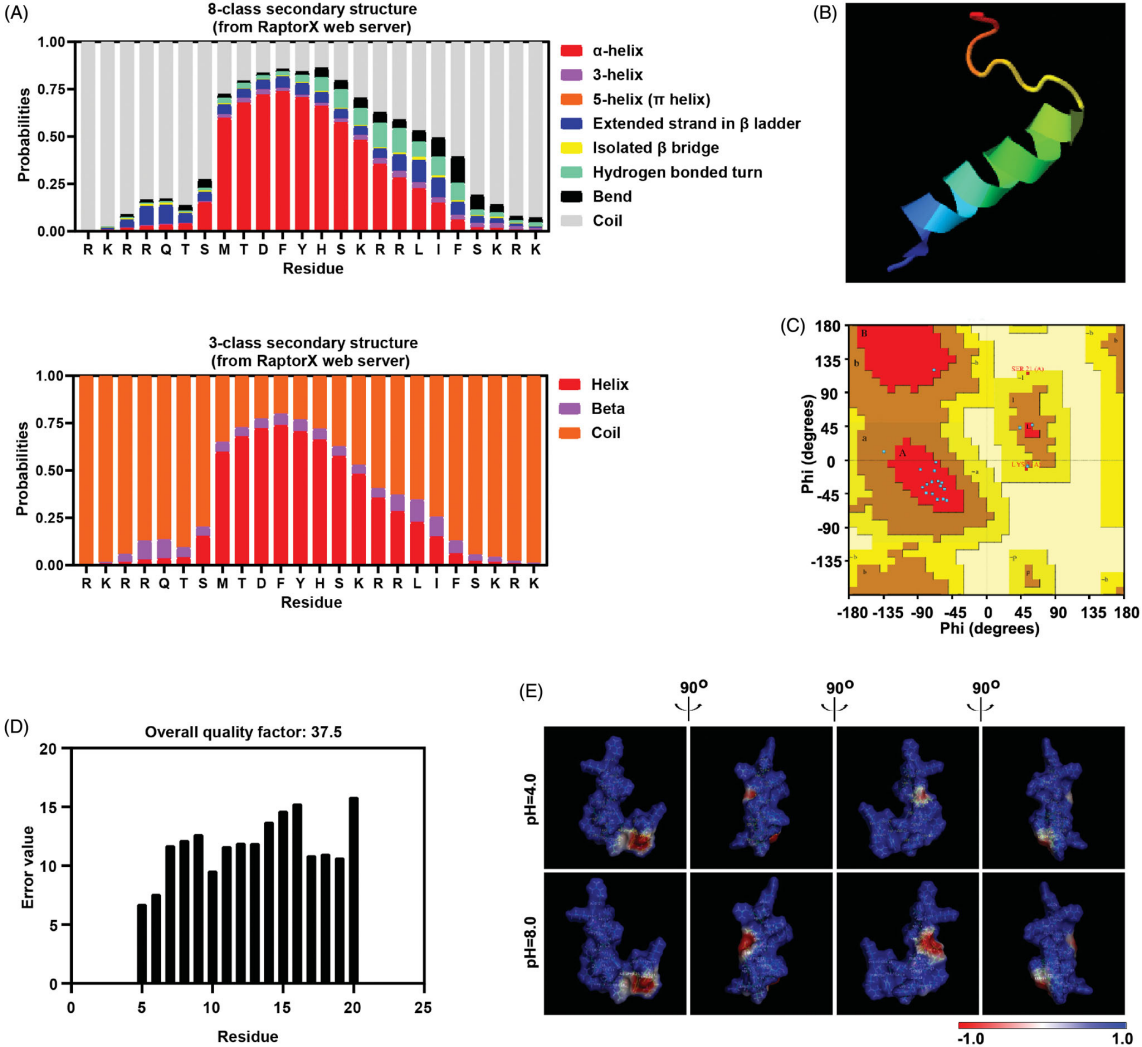
Peptide P2 structure prediction. (A) Secondary structure prediction of peptide P2 was conducted by RaptorX webserver. (B) 3D structural model of peptide P2 predicted by I-TASSER based algorithms web server. (C) Ramachandran plot of peptide P2 modeled by I-TASSER web server. (D) Overall quality of predicted P2 structure checked by ERRAT analysis. (E) Molecular electrostatic potential illustrations of P2. Charge distribution across the peptide P2 surface from different angles are colored according to the intensity of electrostatic potential, stick diagrams of peptide P2 are buried within the semitransparent electrostatic surface maps, colored scale described their charge intensity (negative in red, positive in blue).

**Figure 2.**
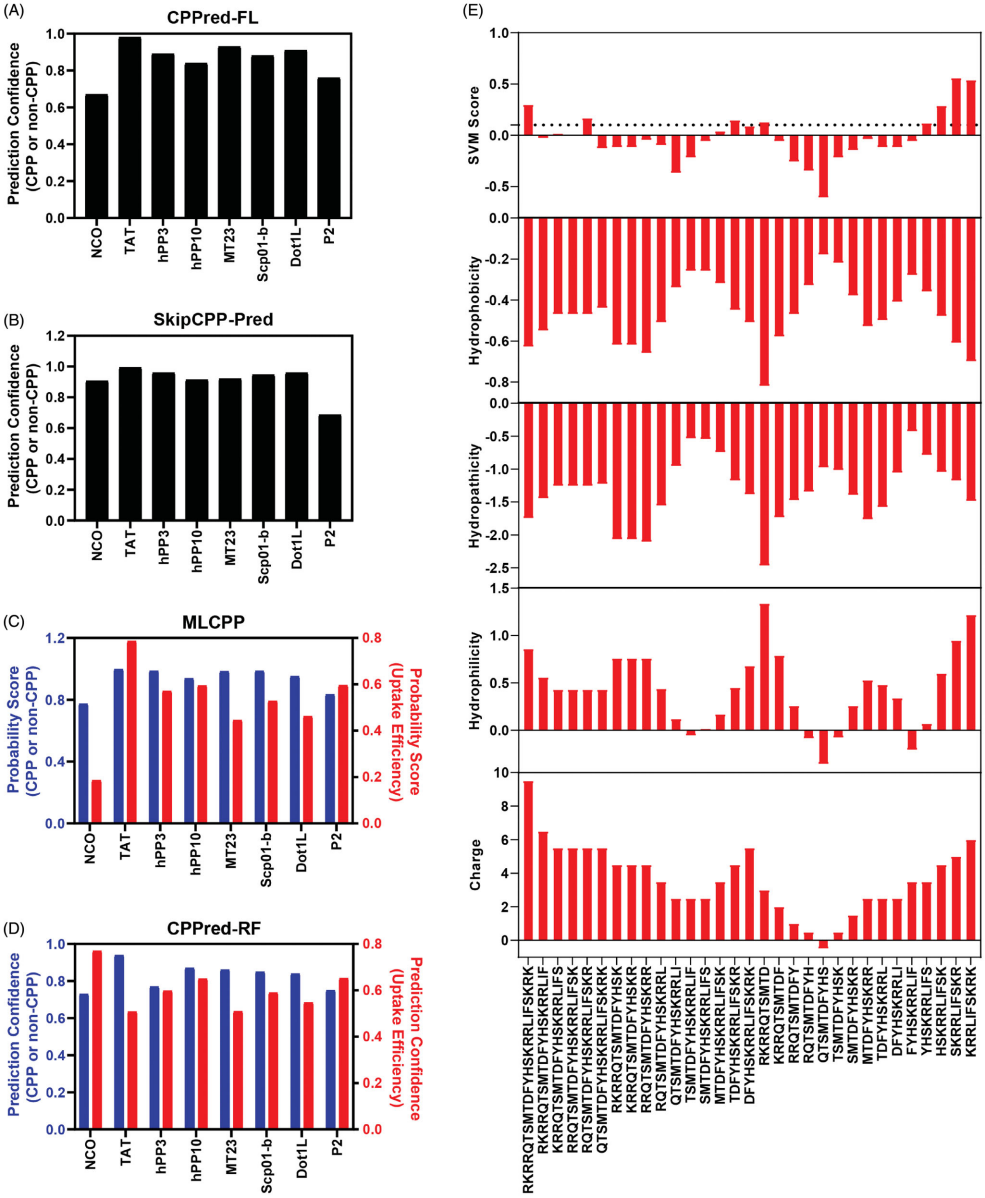
Cell penetrating peptide prediction. (A) CPP or non-CPP prediction of peptide P2 by CPPred-FL server. (B) CPP or non-CPP prediction of peptide P2 by SkipCPP-Pred server. (C) CPP or non-CPP and uptake efficiency prediction of peptide P2 by MLCPP server. (D) CPP or non-CPP and uptake efficiency prediction of peptide P2 by CPPred-RF server. (E) CPP prediction by using physiochemical properties-based SVM model and motif-based model.

### 3.2. Peptide P2 structure prediction

Several reports have suggested that structural properties control the cellular uptake mechanism of CPPs through membrane interactions (Eiríksdóttir et al., 2010). Secondary structure is the general 3D form of the local peptide segment. Here, 8-class secondary structure (Figure 1(A)), 3-class secondary structure (Figure 1(A)), 8-class transmembrane topology (Figure S3(A)), and solvent accessibility (Figure S3(B)) were predicted by the community-wide web-based RaptorX server. Residues 8 to 19 of peptide P2 have a higher probability to form alpha-helix but a relatively lower probability of beta-sheet. This result is consistent with the 3D model structures obtained from I-TASSER (Figure 1(B)), as well as predicted by the NetSurfP server (Figure S3(C)). Both Z‐score (Figure S3(D)) reached −0.11 in the plot of overall model quality evaluation. Local model quality and knowledge-based energy (Figure S3(E)) indicated the good quality of the initial model prediction. Ramachandran plot (Figure 1(C)) suggested that the model of prediction shown above has good quality as well, and ERRAT analysis revealed overall quality factors reaching 37.5 (Figure 1(D)). Furthermore, we examined the electrostatic potential surfaces by the Adaptive Poisson-Boltzmann Solver (APBS) package (Figure 1(E)). The electrostatic surface map also indicated a large positively charged surface area distribution from different angles. Although the immunogenicity of peptide P2 is weaker than previous reported peptide TAT (Wang et al., 2010) and MT23 (Zhou et al., 2017) (Figure S3(F)) predicted by Class I Immunogenicity server, the half-life of peptide P2 in mammalian predicted by ProtLifePred web server is shorter than (Figure S3(F)) known peptide Dot1l, TAT, hPP3, hPP10 and MT23.

**Figure 3.**
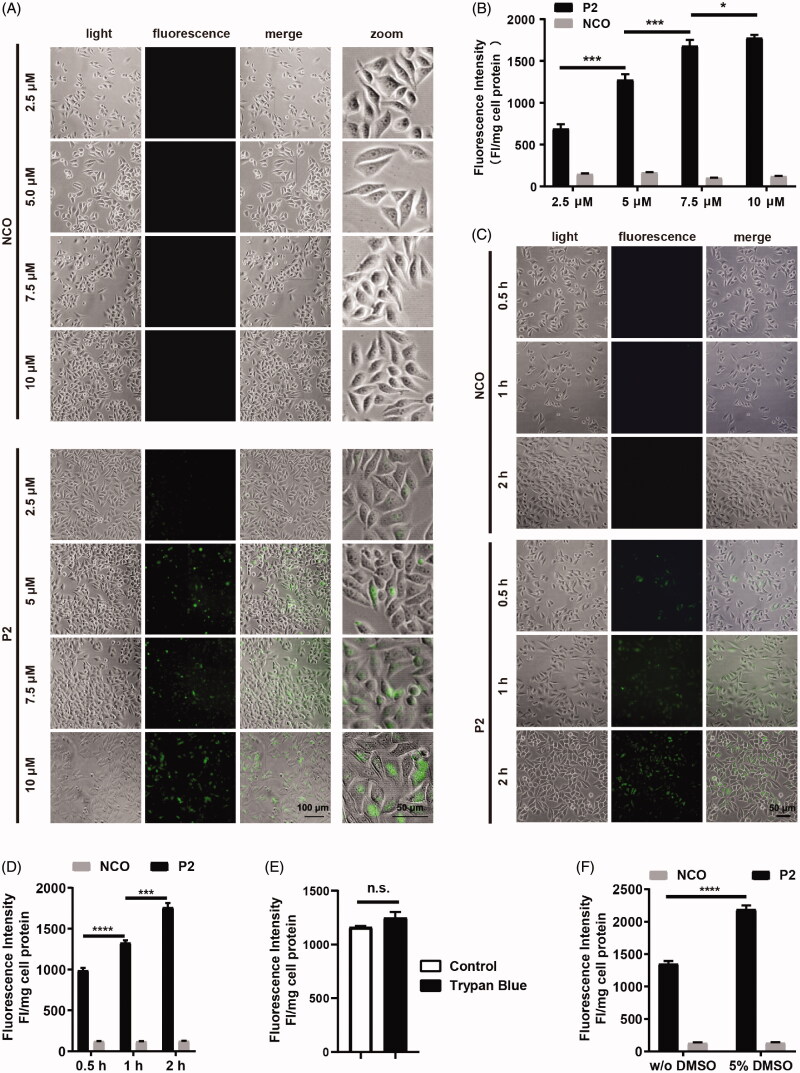
Cellular uptake of peptide P2. (A) Fluorescence microscopy images of FITC-labeled peptide P2 at indicated concentrations for 1 h. (B) Fluorescence intensity quantification of FITC-labeled peptide P2 at indicated concentrations for 1 h. Fluorescence intensity was normalized by protein concentration of cell lysate. All measurements (3 replications of each group) were normalized to the protein concentration of cell lysate, and error bars represent S.E.M., the one‐way analysis of variance (ANOVA) with Tukey–Kramer’s post hoc test was used to compare the differences. (C) Fluorescence microscopy images of FITC-labeled peptide P2 (5 µM) incubation with different time point. (D) Fluorescence intensity quantification of FITC-labeled peptide P2 (5 µM) incubation with different time points. Fluorescence intensity was normalized by protein concentration of cell lysate. All measurements (3 replications of each group) were normalized to the protein concentration of cell lysate, and error bars represent S.E.M., the one‐way analysis of variance (ANOVA) with Tukey–Kramer’s post hoc test was used to compare the differences. (E) Fluorescence intensity quantification of FITC-labeled peptide P2 (5 µM) with or without trypan blue incubation for 1 h. All measurements (3 replications of each group) were normalized to the protein concentration of cell lysate, and error bars represent S.E.M., the Student *t*-test was used to compare the differences. (F) Fluorescence intensity quantification of FITC-labeled peptide P2 (5 µM) incubated with or without 5% DMSO treatment for 1 h. All measurements (3 replications of each group) were normalized to the protein concentration of cell lysate, and error bars represent S.E.M., the Student *t*-test was used to compare the differences.

We also used the PPM server and CellPM server to predict the interaction between the peptide and cell membrane. Residues 12, 15-16, 18 and 24 of peptide P2 may embed into lipid bilayers (Figure S4(A)), and the transfer energy Δ*G*(*z*) of a peptide from water to DOPC bilayer is shown in Figure S4(B). This peptide-membrane interaction prediction suggested that the optimal translocation pathway of the peptide is through the lipid bilayer, which reflects peptide P2’s affinity to different membrane regions. Circular dichroism spectroscopy was also performed to further validate the secondary structure of peptide P2 (Figure S5(A)), although a weak α‐helix structure was formed in a low concentration of phosphate-buffered saline. Lastly, the aggregation propensity of peptide P2 was characterized by native PAGE, a single band of P2 and without apparent aggregation was observed in Figure S5(B).

**Figure 4.**
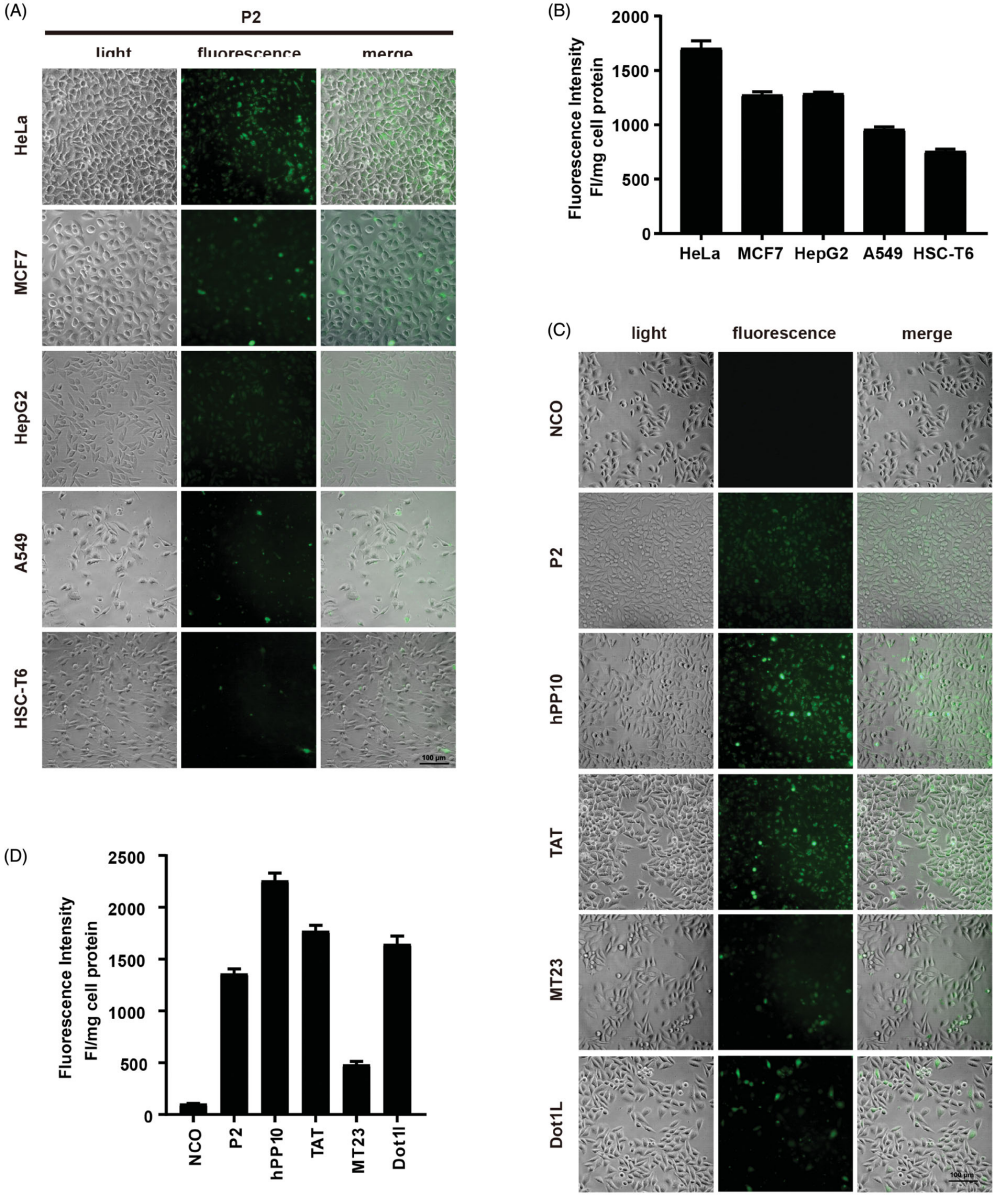
Cellular uptake of peptide P2 under different conditions. (A) Fluorescence microscopy images of FITC-labeled peptide P2 (5 µM) in different cell lines for 1 h. (B) Fluorescence intensity quantification of FITC-labeled peptide P2 (5 µM) in different cell lines for 1 h. All measurements (3 replications of each group) were normalized to the protein concentration of cell lysate, and error bars represent S.E.M., the one‐way analysis of variance (ANOVA) with Tukey–Kramer’s post hoc test was used to compare the differences. (C) Fluorescence microscopy images of FITC-labeled peptides (5 µM) other than peptide P2 for 1h. (D) Fluorescence intensity quantification of FITC-labeled peptides (5 µM) other than peptide P2 for 1 h. All measurements (3 replications of each group) were normalized to the protein concentration of cell lysate, and error bars represent S.E.M., the one‐way analysis of variance (ANOVA) with Tukey–Kramer’s *post hoc* test was used to compare the differences.

**Figure 5.**
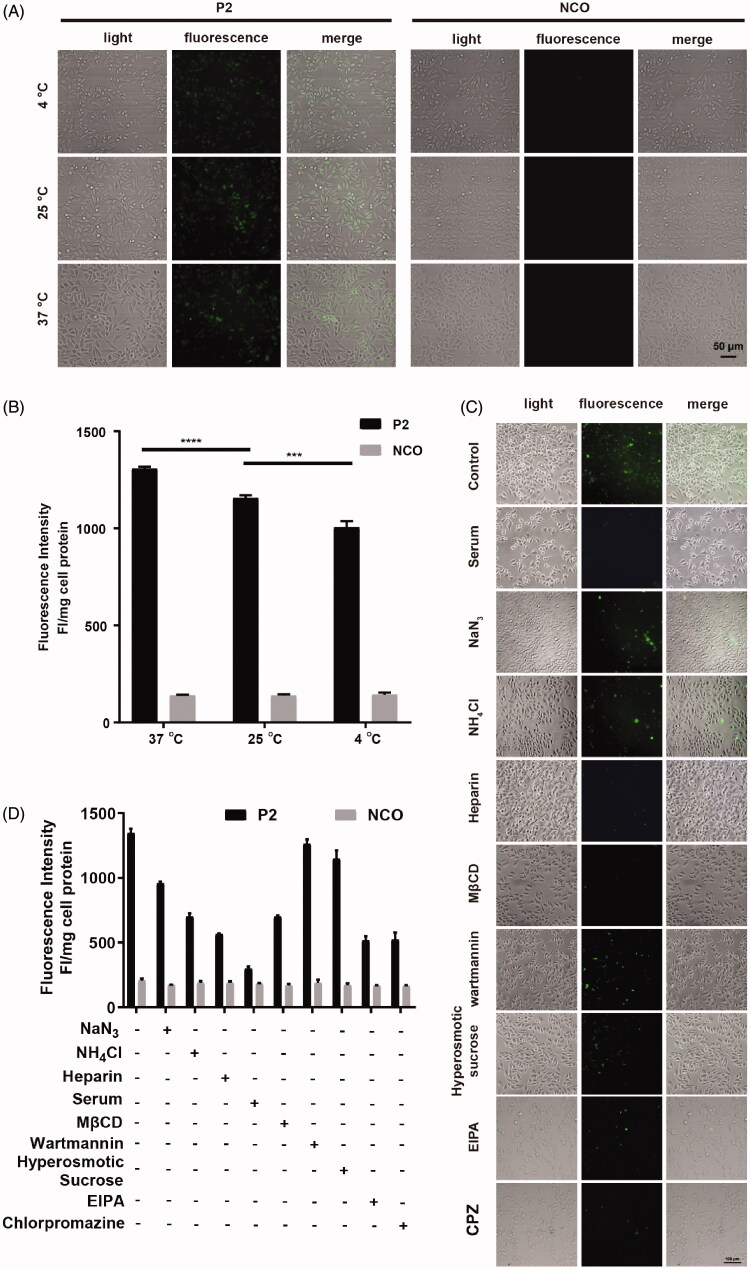
Involved pathway of cellular uptake of peptide P2. (A) Fluorescence microscopy images of FITC-labeled peptide P2 (5 µM) at different temperature for 1 h. (B) Fluorescence intensity quantification of FITC-labeled peptide P2 (5 µM) at different temperature for 1 h. All measurements (3 replications of each group) were normalized to the protein concentration of cell lysate, and error bars represent S.E.M., the one‐way analysis of variance (ANOVA) with Tukey–Kramer’s *post hoc* test was used to compare the differences. (C) Fluorescence microscopy images of FITC-labeled peptide P2 (5 µM) exposed with different inhibitors for 1 h. (D) Fluorescence intensity quantification of FITC-labeled peptide P2 (5 µM) exposed with different inhibitors for 1 h. All measurements (3 replications of each group) were normalized to the protein concentration of cell lysate, and error bars represent S.E.M., the one‐way analysis of variance (ANOVA) with Tukey–Kramer’s *post hoc* test was used to compare the differences.

### 3.3. Penetration property prediction

Conventional wet-lab experimental approaches to evaluate CPP penetration properties are time-consuming and labor-intensive. In the past few years, several bioinformatic tools have become available to predict the cell-penetrating properties of peptides. To better evaluate their penetration properties, the sequences of P2 and existing CPPs, such as TAT, hPP3, hPP10, MT23, Scp01-b, and Dot1l, were assessed by several prediction algorithms (CPPred-FL, SkipCPP-Pred, MLCPP and CPPred-RF). As expected, TAT, hPP3, hPP10, MT23, Scp01-b and Dot1l all have a higher penetration probability than well-known non-CPP NCO sequences. Peptide P2 has a higher probability than NCO as predicted by CPPred-FL (Figure 2(A)), MLCPP (Figure 2(C)) and CPPred-RF (Figure 2(D)), but not in SkipCPP-Pred (Figure 2(B)) prediction. This may result in different sensitivities and accuracies of the prediction server itself. Furthermore, motif prediction information was obtained via a support vector machine (SVM) classifier package. SVM score and various features (hydrophobicity, hydropathicity, hydrophilicity and charge) as SVM input were calculated (Figure 2(E)). Full-length peptide P2 and truncated c-terminal have higher SVM score, which suggests that P2 have the necessary penetration property. Moreover, as shown in Figure 2(C,D), MLCPP and CPPred-RF prediction also provide uptake efficiency. Peptide P2 has a higher penetration efficiency than NCO peptide (Figure 2(C)) but failed in the CPPred-RF prediction model. Therefore, easy-to-implement tools via webserver provide us information and prediction on penetration probability, which can be used to facilitate early-stage CPP screening prior to the wet-lab test.

### 3.4. Penetrating property of peptide P2

To confirm and visualize the intracellular distribution of peptide P2, HepG2 cells were incubated with FITC-labeled peptides for 1 hour at the indicated concentration. Then, we evaluated the penetration property of P2 with fluorescence microscopy. Fluorescence positive cells (Figure 3(A)) and normalized fluorescence intensity (Figure 3(B)) of peptide significantly increased as peptide concentration increased, but there are no changes in scramble peptide NCO. We also examined the penetration efficiency of peptide P2, and both fluorescence microscopy (Figure 3(C)) and normalized fluorescence intensity (Figure 3(D)) significantly increased with incubation time. To further eliminate the fluorescence signal at the cell surface, we used trypan blue to treat cells with peptide P2 following the protocol published (Zhang et al., 2018; Geng et al., 2020), we found that there is no significant difference between trypan blue treated and control group (Figure 3(E)), which suggested that peptide P2 was fully internalized of the cell treated. Moreover, as expected, the penetration efficiency of peptide P2 can be enhanced by 5% DMSO treatment (Figure 3(F)).

Next, we examined the penetration efficiency of peptide P2 in different cell lines. Fluorescence microscopy images suggested that fluorescence in HeLa cells was higher than those in other cell lines including MCF7, HepG2, A549 and HSC-T6. Fluorescence intensity qualification was also consistent with images we captured (Figure 4(B)). Moreover, we also found that peptide P2 can efficiently penetrate hard-to-translocate murine macrophage cell line RAW264.7 (Figure S6(A)) and primary cultured mouse liver parenchymal cells and whole primary cultured hepatocytes (Figure S6(B)). A different peptide with different amino acid compositions may have different penetration efficiencies. Therefore, we examined penetration efficiencies of well-known CPPs, including TAT, hPP10, MT23 and Dot1l. Fluorescence microscopy images (Figure 4(C)) and fluorescence intensity (Figure 4(D)) data suggested that P2 has higher penetration efficiency than MT23 but lower than TAT and hPP10 and Dot1l.

**Figure 6.**
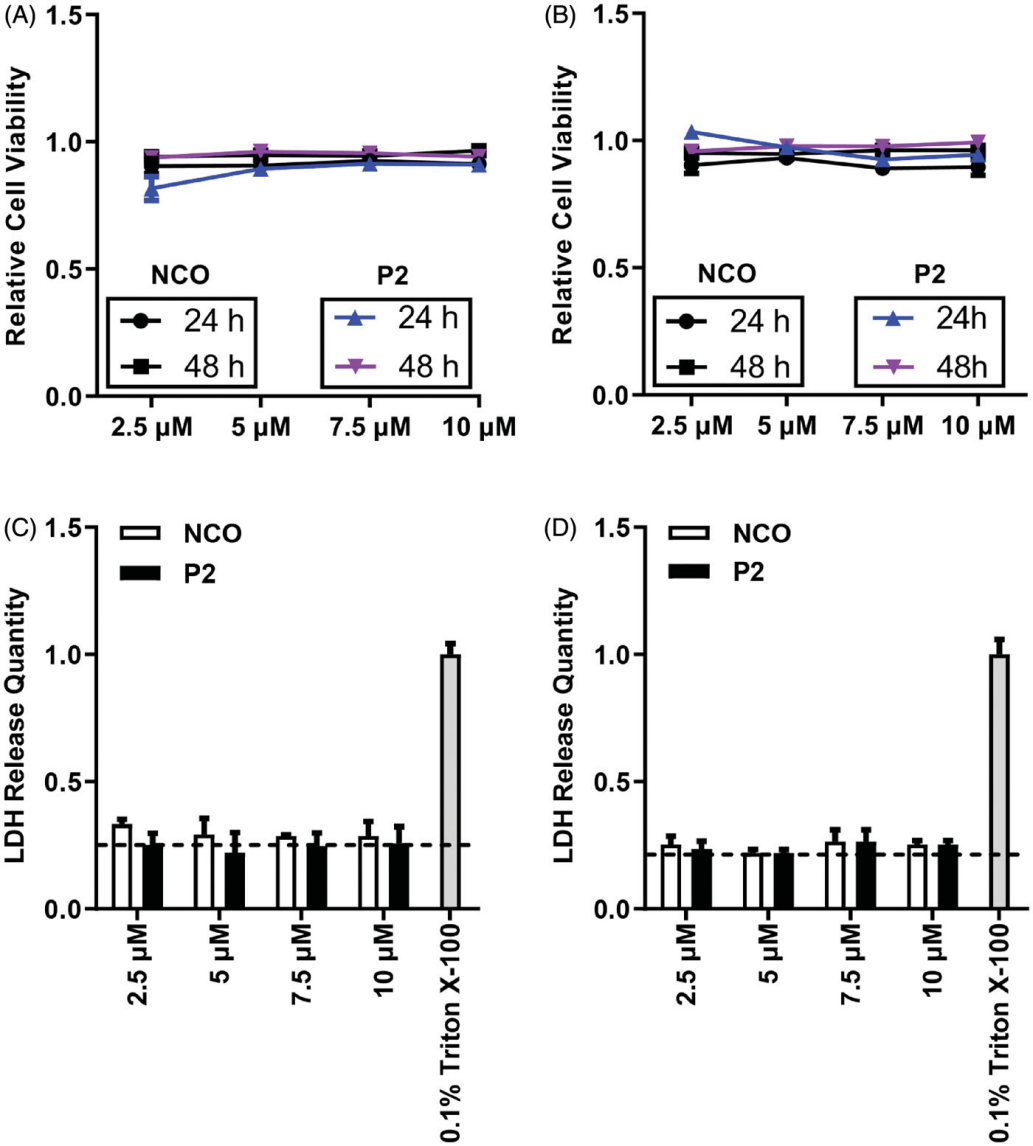
Cytotoxicity evaluation on peptide P2. (A) Cell viability assessment of HeLa cells incubated with the indicated concentration of peptide P2 after 24 and 48 hours. All measurements (3 replications of each group) were normalized to the protein concentration of cell lysate, and error bars represent S.E.M., the two‐way analysis of variance (ANOVA) with Bonferroni’s multiple comparison test was used to compare the differences. (B) Cell viability assessment of HepG2 cells incubated with the indicated concentration of peptide P2 after 24 and 48 hours. All measurements (3 replications of each group) were normalized to the protein concentration of cell lysate, and error bars represent S.E.M., the two‐way analysis of variance (ANOVA) with Bonferroni’s multiple comparison test was used to compare the differences. (C) LDH release assay of HeLa cells incubated with the indicated concentration of peptide P2, 0.1% Triton X-100 was used as a positive control. The baseline indicated in the plot represents negative control. (D) LDH release assay of MCF7 cells incubated with the indicated concentration of peptide P2, 0.1% Triton X-100 was used as a positive control. The baseline indicated in the plot represents negative control.

### 3.5. Penetrating property of peptide P2 under different conditions

In addition to peptide concentration, incubation time and cell type, factors like penetration mechanism, temperature, and internalization pathway are also critical to the penetrating property of peptide P2. Among these factors, the temperature has been noted as a factor to influence CPP permeation by increasing cellular metabolism, rigidity and phase state (Toyohara et al., 2019). Therefore, we firstly investigated the effect of temperature on CPP penetration efficiency. In Figures 5(A,B), fluorescence microscopy images and normalized fluorescence intensity quantifications suggest that the penetrating efficiency of peptide P2 is temperature dependent, which is consistent with previous reports (Vivès et al., 2003). Based on this study and in reference to our result, we, therefore, speculate that higher temperature results in high endocytosis and less rigid membrane thus allowing the highly efficient entry of peptide P2. Since temperature is a key environmental factor that impacts cellular endocytosis, we examined the role of cellular endocytosis in the penetration property of peptide P2. To characterize the mechanism of uptake of peptide P2, cellular internalization inhibitors NaN_3_ (metabolic inhibitor (Wang et al., 2010)), NH_4_Cl (lysosomal pH-neutralizing agent (Qiang et al., 2018)), Heparin (soluble analog of heparin sulfate proteoglycans that inhibits endocytosis (Wang et al., 2016a; Geng et al., 2020)), Serum, MβCD (lipid raft-mediated endocytosis inhibitor (Zhang et al., 2019)), Wortmannin (receptor-mediated endocytosis inhibitor of blocking PI-3 kinase (Geng et al., 2020)), hyperosmotic sucrose (clathrin-dependent endocytosis inhibitor (Wang et al., 2016c)), Chlorpromazine (clathrin-mediated endocytosis inhibitor (Park et al., 2019)), and EIPA (5-(N-ethyl-N-isopropylamiloride, macropinocytosis inhibitor (Elmquist et al., 2006)) were employed to treat cells incubated with peptide P2. As shown in Figure 5(C,D), endocytosis-related inhibitors can significantly decrease the penetration efficiency of peptide P2. Therefore, endocytosis pathways, including receptor-mediated endocytosis, may involve cellular uptake of peptide P2, although the penetration of peptide P2 was slightly blocked by wortmannin and hyperosmotic sucrose.

### 3.6. Cytotoxicity assessment of peptide P2

To assess the cytotoxicity of peptide P2 to the cell, MTT assay, LDH assay, and hemolysis analysis were performed on HepG2 and HeLa cells, as shown in Figure 6. In the MTT assay, HeLa (Figure 6(A)) and HepG2 (Figure 6(B)) cells were treated with indicated concentrations (from 2.5 to 100 µM) of peptide P2 for 1 hour, and no significant cell growth changes were observed in HeLa cells (Figure 6(A)). The cell growth of HepG2 was slightly affected in 24 hours but was back to normal in 48 hours (Figure 6(B)). In the LDH assay, we did not observe any damages to the HeLa (Figure 6(C)) and MCF7 (Figure 6(D)) cell membrane before and after peptide P2 treatment. Moreover, we did not observe red blood cell membrane damage on classic hemolysis assay after peptide P2 treatment (Figure S7). Therefore, these data suggest that penetration by peptide P2 is safe, and membrane permeability of peptide P2 does not mediate cell membrane disturbance.

**Figure 7.**
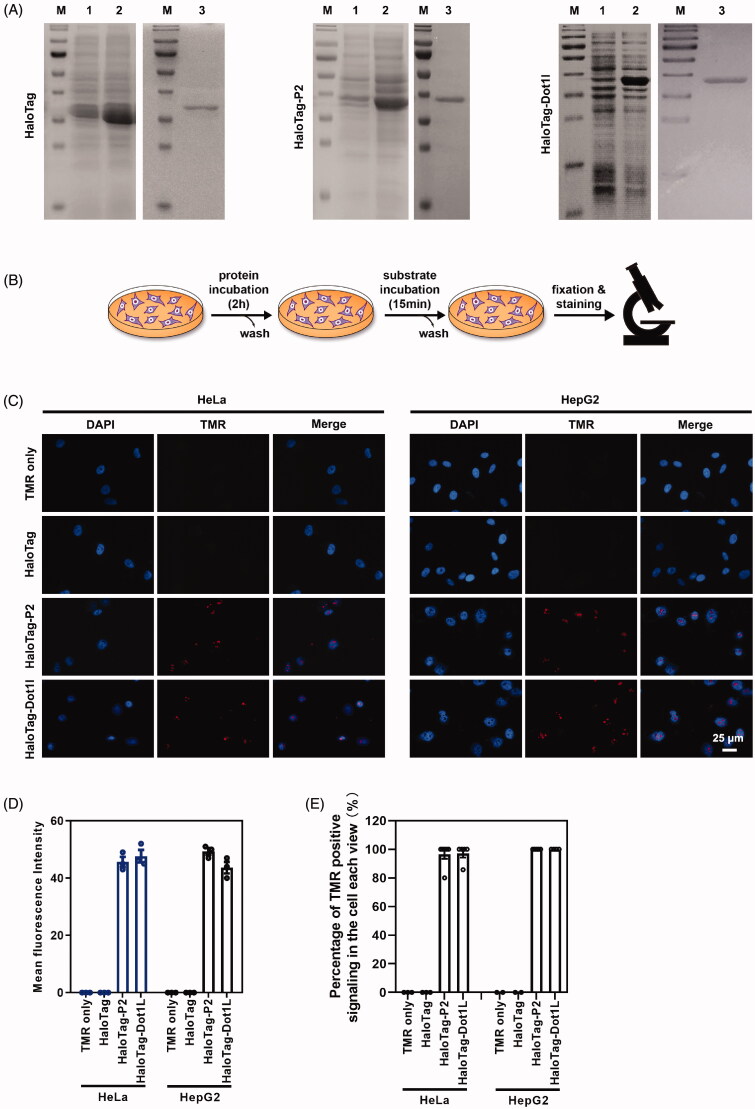
Peptide P2 mediate HaloTag delivery. (A) HaloTag, HaloTag-P2 and HaloTag-Dot1l protein preparation and purification. Lane 1 indicated non-induction, Lane 2 indicated IPTG induction, and Lane 3 indicated purified protein. (B) Schematic diagram of the protein treatment protocol and assessment thereafter. (C) Fluorescence microscopy images of peptide P2 (0.25 µg/ml) mediated HaloTag delivery for 2 h. (E) Quantification of fluorescence intensity of peptide P2 (0.25 µg/ml) mediated HaloTag delivery in the cell for 2 h. All measurements (3 replications of each group) were normalized to the protein concentration of cell lysate, and error bars represent S.E.M., the one‐way analysis of variance (ANOVA) with Tukey–Kramer’s *post hoc* test was used to compare the differences. (D) Percentage of TMR positive cells treated with proteins.

### 3.7. Peptide P2 delivered HaloTag for high sensitivity imaging

HaloTag, a type of self-labeling protein tag, is a modified haloalkane dehalogenase derived from a bacterial enzyme. Containing 297 amino acids (33 kDa) (England et al., 2015), it cannot pass through the cell membrane without a transport vector. Data shown in this paper suggest that peptide P2 is an alternative CPP for potential macromolecule delivery. We constructed the HaloTag-P2 fusion expression plasmid, and we induced its expression and purified fusion protein via a prokaryotic protein expression system (Figure 7(A)). We also prepared protein HaloTag and HaloTag-Dot1l (a CPP found by our group (Geng et al., 2020)) as control (Figure 7(A)). Then, we followed our protocol shown in Figure 7(B) to treat HepG2 and HeLa cells. After substrate TMR incubation and washing, fluorescence images were captured. As shown in Figure 7(C,D), Figure S8(A–C), a very low amount of (0.25 µg/ml or 0.4 µg/ml protein) peptide P2 fused with HaloTag can be delivered into intracellular and cell nucleus, but the groups of TMR substrate only and HaloTag protein only do not have any signaling in the cell. These results suggest that peptide P2 not only can penetrate culture cell lines but also can deliver macromolecules like self-labeling HaloTag into cells.

## 4. Discussion

A variety of drugs act on disease-associated targets located within the eukaryotic cytoplasm or nucleus. Phospholipid bilayer membrane is a practically impermeable barrier, and it controls ions and particles between the inner cell and external microenvironment (Liu et al., 2016; Wu et al., 2018). Therapeutic peptides and proteins need to gain access to the cytosol or nucleus prior to exerting their functions, and many routes of administration have been tried by investigators worldwide for their effective delivery. A group of peptides called cell-penetrating peptides (CPPs), which were found to have the ability to transport different types of cargo molecules across the phospholipid bilayer membrane, have been widely applied in the treatment of various diseases in preclinical studies (Liu et al., 2016). Due to the many biomedical applications of CPPs, especially in drug delivery, the identification of novel and highly efficient CPPs is extremely urgent. However, identification and screening of highly efficient CPPs are often tedious. Once screening and identification have been completed, every peptide must be examined for its potential cell-penetrating activity, which is a time-consuming and laborious cycle (Manavalan et al., 2018). Few papers (Hu et al., 2020; Kardani and Bolhassani, 2021) published showed that bioinformatic tools were used to predict CPP, however, they did not predict the CPP systematically such as CPP prediction, penetration efficiency, structural, peptide-membrane interaction, physical-chemical properties, and wet-lab experiment validation. We found that peptide P2 has higher penetration efficiency than MT23 but lower than TAT, hPP10 and Dot1l. To improve the penetration efficiency of peptide P2, we can use CPP and CPP penetration efficiency prediction tools to compare the potential efficiency of peptides with different mutations, after that, wet-lab experiments can be performed to validate the penetration efficiency of the highest candidate. Furthermore, other computational approaches that combine structural, peptide-membrane interaction and physical-chemical prediction can be used to evaluate the property of the candidate and redesign the scaffold sequence.

Because of their flexibility, the structures of peptides are difficult to study experimentally. The aim of the present study is to identify a new cell-penetrating peptide, P2. We combined bioinformatic analysis of peptide P2 at the physio-chemical level, secondary structure, and 3D structure level, as well as CPP or non-CPP prediction through the webserver. This prediction pipeline took advantage of the physicochemical properties of the peptide. Specifically, we compared the prediction scores and probabilities, employed algorithms to reevaluate prediction parallelly. This study will assist peptide researchers in selecting appropriate prediction tools that best suit their purposes. High-throughput screening of functional peptides like cell-penetrating peptides can accelerate the development of drug delivery systems. However, the different webservers may still have some limitations (listed in Table 1) such as the small number of peptide datasets used to train the algorithm (Feger et al., 2020). We, therefore, selected combined web-accessible approach to design CPP, which can assist the design of efficient and safe CPP for drug delivery.

In this study, a short peptide derived from CDN1 protein with a higher proportion of basic residues was identified. We found that peptide P2 can efficiently enter different cell lines through a concentration-dependent manner. The endocytosis pathway, especially receptor-related endocytosis, may be involved in the process of P2 penetration. Our data also show that peptide P2 is safe in cultured cell lines and red blood cells.

Impermeable HaloTag protein or its expression plasmid generally cannot across the membrane automatically (Liu et al., 2010; Wang et al., 2014), thus leading to the HaloTag-based labeling unfree. Peptide P2 can highly effectively deliver self-labeling protein HaloTag into cells, which can be used to image in a sequential model using appropriate substrates, such as TMR, Oregon Green, and Alexa Fluor® 488 and 660 to monitor cell status and function. Before the HaloTag-P2 labeling system was applied as a new potential tool for cell sorting, cell imaging and other cell biological applications, the key points in application of this methodology to future such as in vivo transduction efficiency evaluation, tissue distribution, targeting and stability need to be carefully addressed.

In summary, we identified a new cell-penetrating peptide P2 derived from CDN1 through *in silico* identification and experimental validation of penetration property, and peptide P2 can efficiently facilitate self-labeling protein’s functional delivery to cells.

## Supplementary Material

Supplemental Material
