# Supplementary material for: In silico identification and experimental validation of cellular uptake and intracellular labeling by a new cell penetrating peptide derived from CDN1
Source: Drug Deliv. 2021 Aug 31;28(1):1722–36. doi: 10.1080/10717544.2021.1963352 (PMC8409945; doi:10.1080/10717544.2021.1963352)
Supplement: Supplemental Material [file IDRD_A_1963352_SM3074.docx]

Supporting information

In silico identification and experimental validation of cellular uptake and intracellular labelling by a new cell penetrating peptide derived from CDN1

Xiangli Guo^1,2^, Linlin Chen^2,3^, Lidan Wang^1,2^, Jingping Geng^1,2^, Tao Wang^4^, Jixiong Hu^5^, Jason Li^6^, Changbai Liu^2*^, Hu Wang^1, 10*^

^1^ Department of Pathology and Immunology, Medical School, China Three Gorges University, Yichang 443002, China;

^2^ Hubei Key Lab. of Tumor Microenvironment and Immunotherapy, China Three Gorges University, Yichang, 443002, China;

^3^ Affiliated Ren He Hospital of China Three Gorges University, Yichang 443002, China;

^4^ The first clinical medical college of China Three Gorges University, Yichang 443002, China;

^5^ College of Life Science, Yangtze University, Jingzhou 434000, China;

^6^ Department of Biology, Johns Hopkins University, Baltimore, MD 21210, USA.

^10^ Lead Contact

*Correspondence:

Changbai Liu, MD, PhD, Hubei Key Lab of Tumor Microenvironment and Immunotherapy, China Three Gorges University, 443002 Yichang, China. Email: cbliu@ctgu.edu.cn.

Hu Wang, PhD, Department of Pathology and Immunology, Medical School, China Three Gorges University, Yichang 443002, China. Email: biomed_wang@yahoo.com.

**Supplementary figure legends**

**
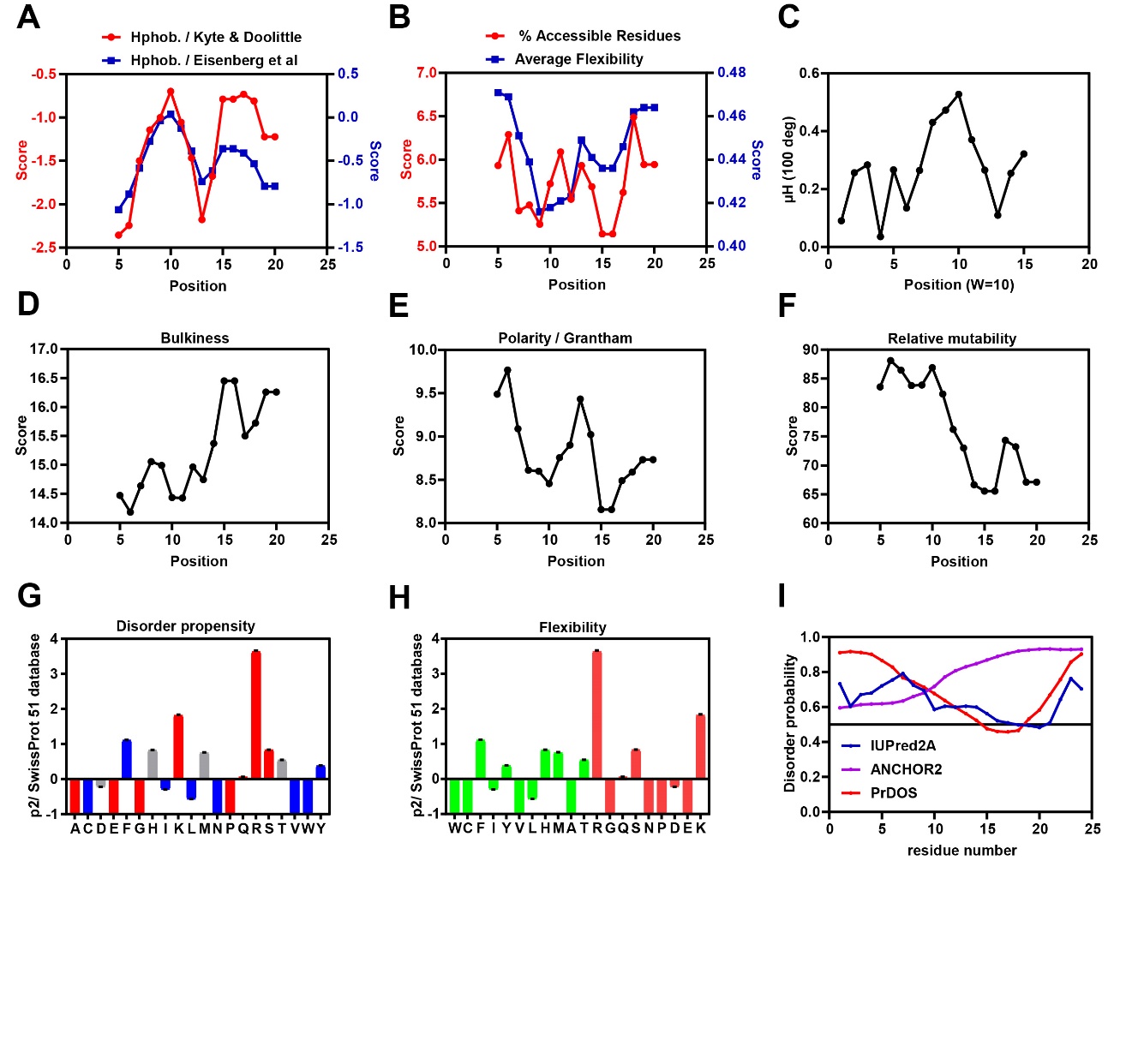
 Figure S1 The hydrophilicity, polarity, flexibility parameters predicted by ExPASy ProtScale. X-axis represents peptide P2 length from N- to C-terminal. Y-axis reveals the score calculated by relative algorithm. The high score represents high probability.**

1. Hydrophobic property of peptide P2 was examined by Kyte & Doolittle and Eisenberg et al method. Graph plot reveals the average hydropathy of entire window indicated on the x-axis corresponding amino acids of peptide P2.
2. Plot reveals the percentage of accessible residues and average flexibility of peptide P2.
3. Hydrophobic moment profiles for peptide P2 were calculated using default angle of 100 degrees.
4. Bulkiness plot for the side chains of peptide P2.
5. Polarity plot for the side chains of peptide P2.
6. Relative mutability of peptide P2.
7. Peptide P2 amino acid composition features relevant to disorder propensity. Analysis was conducted using the SwissProt 51 database and Composition Profiler. Peptide P2 residues are displayed on the x-axis in alphabetical order, disorder propensity of residues is also colored (disordered promoting residues shown in red, order promoting residues shown in blue, and order-disorder neutral residues shown in gray).
8. Peptide P2 composition features relevant to flexibility. Peptide P2 residues are displayed on the x-axis and ordered based on peptide flexibility (rigidity promoting residues are displayed in green and flexibility promoting residues are displayed in red).
9. Probability prediction of disordered regions of P2 analyzed by using IUPred2A (blue), ANCHOR2 (purple), and PrDOS (red) algorithms.

**
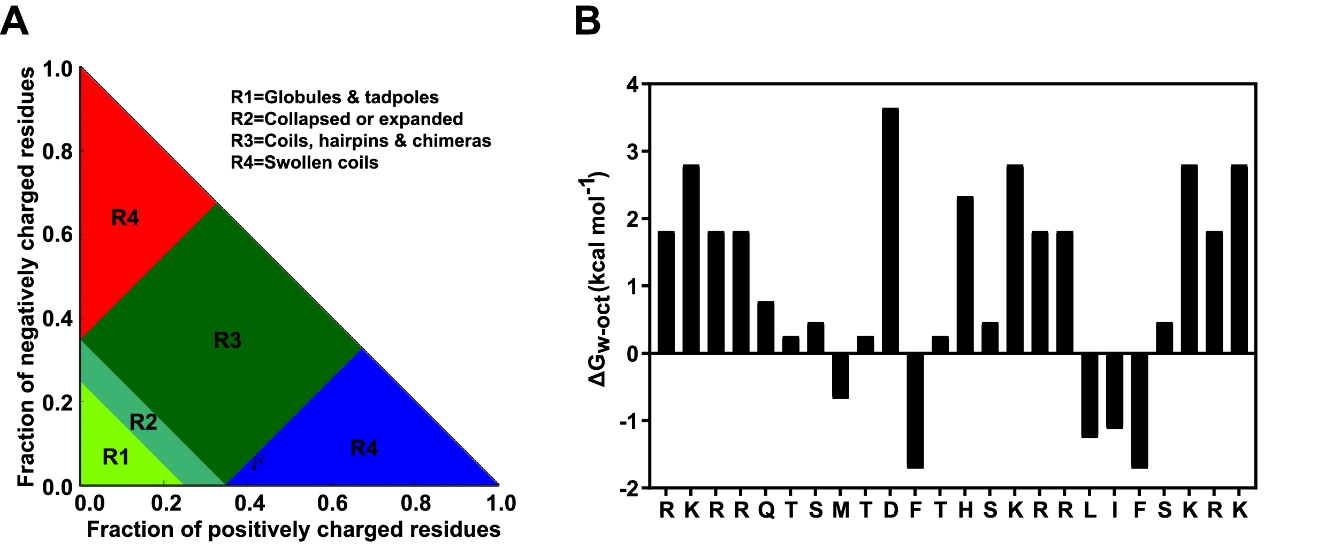
**

**Figure S2 Primary structure analyses of peptide P2**

1. Das–Pappu plot of P2, P2 location is indicated by black dot in Region R4.
2. Whole-residue Wimley–White hydrophobicity indices per amino acid residue plot.

**
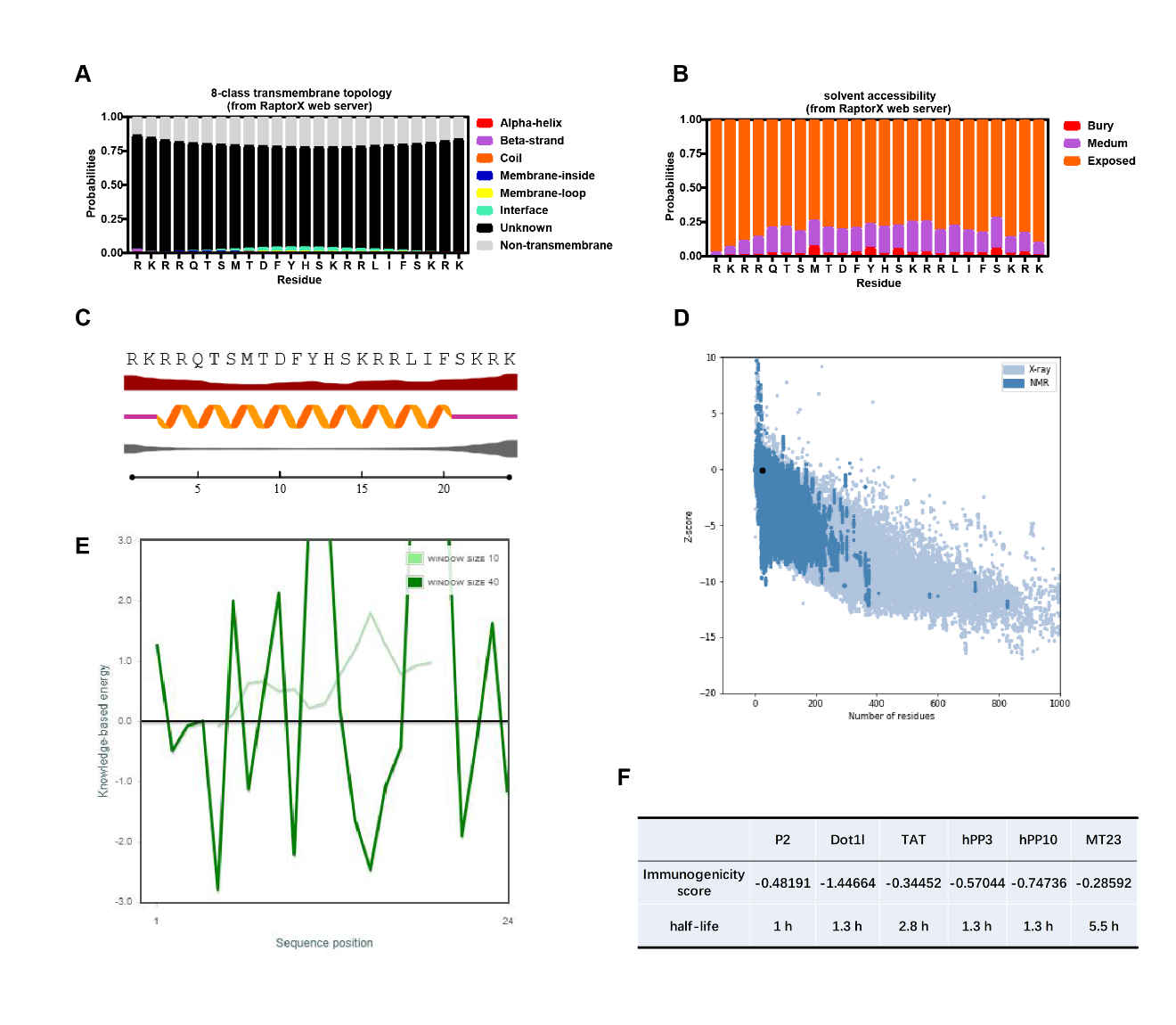
**

**Figure S3 Peptide P2 structure prediction and validation**

1. Transmembrane topology was predicted by RaptorX web server.
2. Solvent accessibility was predicted by RaptorX web server.
3. Relative surface accessibility, secondary structure, and disorder of peptide P2 was predicted by NetSurfP server. From top panel to bottom panel, top panel shows P2 residues, the second panel shows relative surface accessibility (red indicated as exposure possibility, threshold at 25%), the third panel shows secondary structure (orange indicated as alpha-Helix, pink indicated as Coil), the fourth panel shows disorder propensity (thickness of line indicated disordered probability), and the bottom panel show the number of residues.
4. The Z‐score plot of peptide P2 evaluated by ProSA-web program.
5. Local model quality/knowledge-based energy plot of peptide P2 evaluated by ProSA-web program.
6. Immunogenicity and half-life prediction of peptide P2.

**
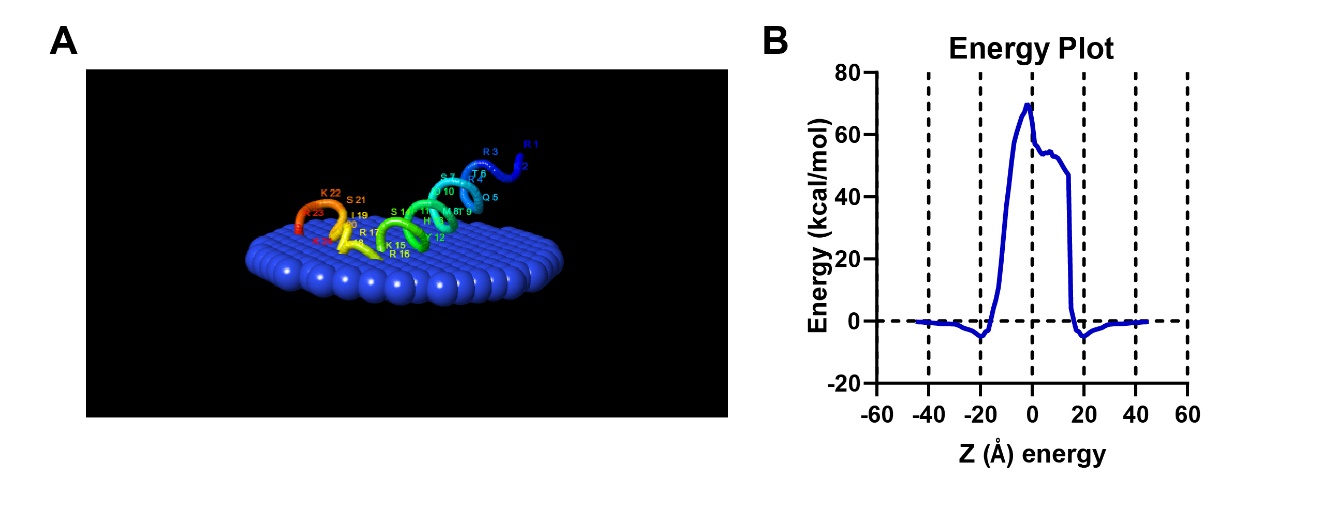
**

**Figure S4 Peptide-membrane interaction prediction**

1. Peptide P2 membrane interaction prediction by PPM web server.
2. Peptide P2 membrane interaction prediction by CellPM web server.

**
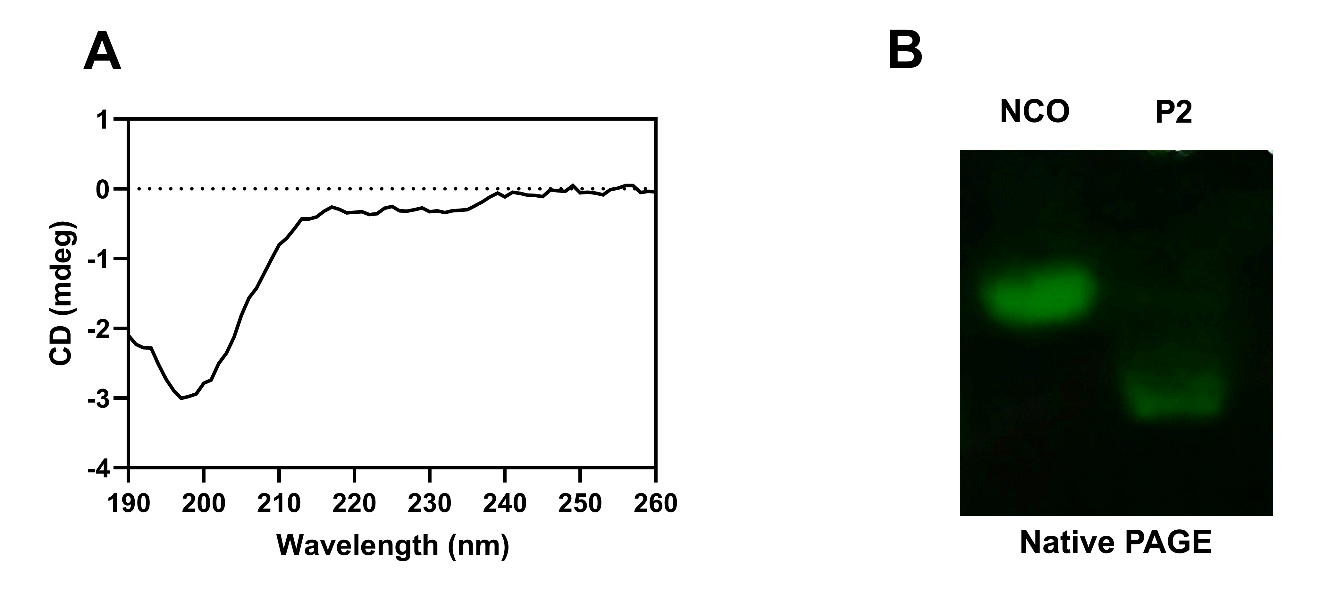
**

**Figure S5 Structure determination and aggregation evaluation**

1. Circular dichroism spectroscopy of peptide P2 (0.18 mg/ml) in phosphate buffer.
2. Native PAGE of peptide, 4 µl of P2 (500 µM) and NCO (500 µM) were loaded.

**
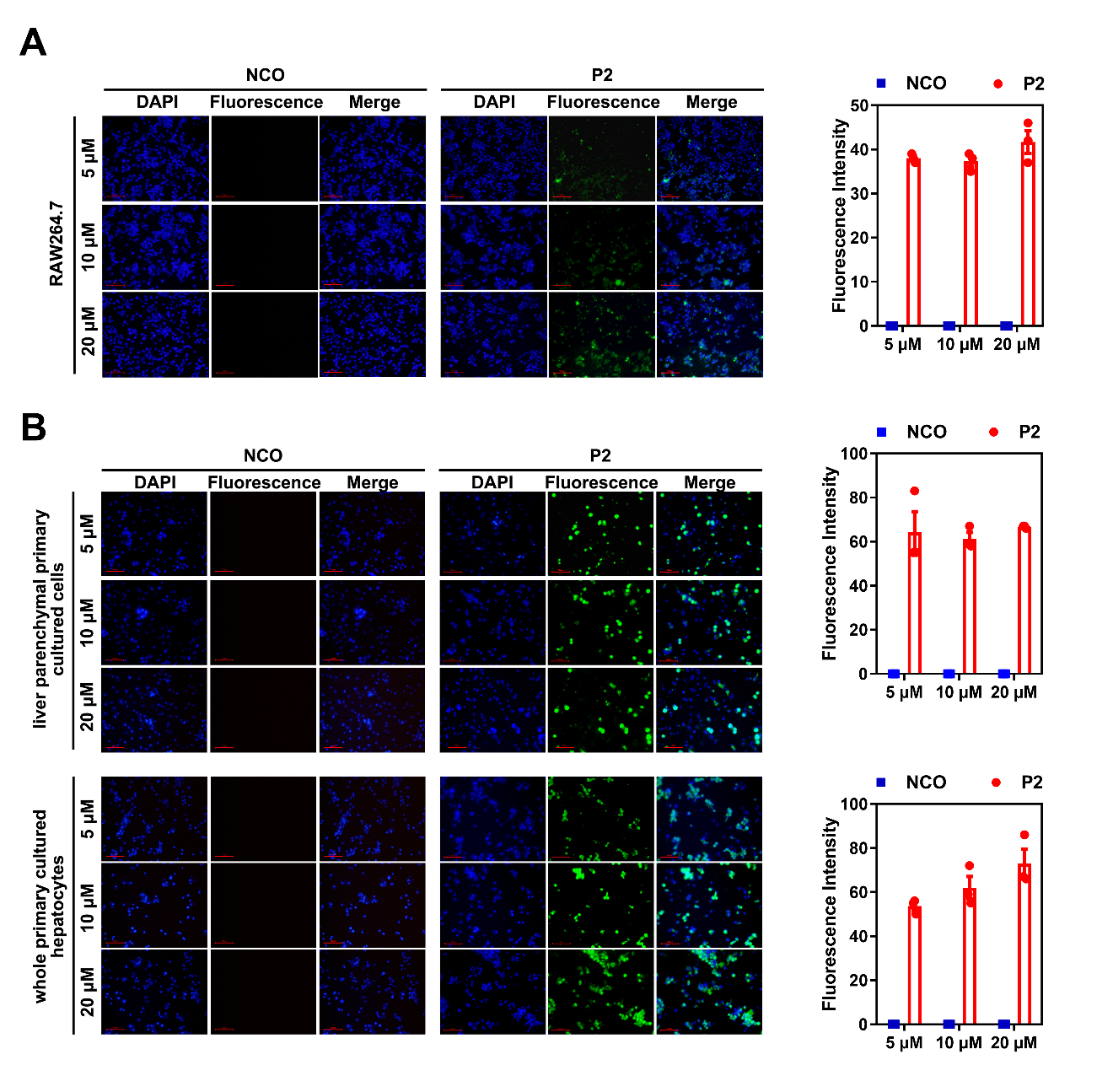
**

**Figure S6 Penetration of P2 in RAW264.7 and primary cultured cells**

1. Left panel showing the penetration efficiency of P2 (5-20 µM) for 1 h treatment in RAW264.7 examined by fluorescence microscope; right panel show the quantification of penetration efficiency of peptide P2.
2. Left panel showing the penetration efficiency of P2 (5-20 µM) for 1 h treatment in primary cultured liver parenchymal cells and whole primary cultured hepatocytes examined by fluorescence microscope, right panel show the quantification of penetration efficiency of peptide P2 in primary cultures.

**
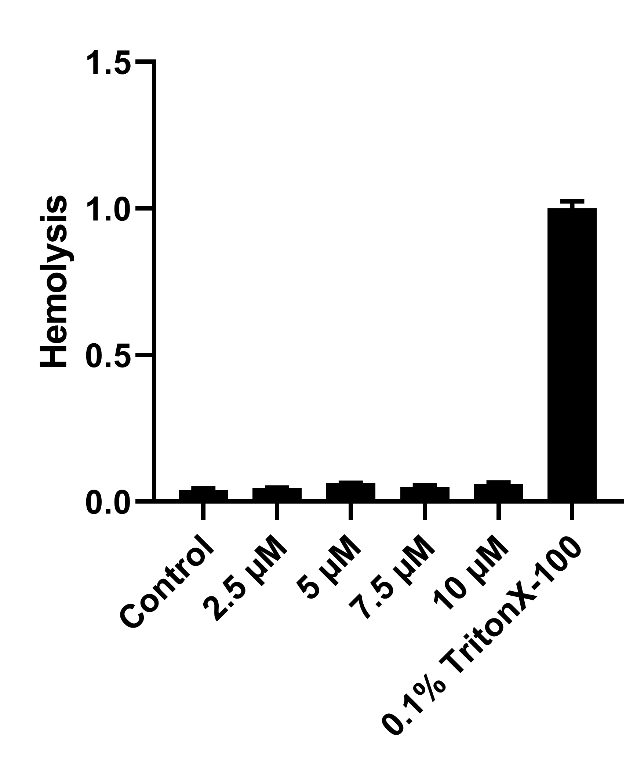
**

**Figure S7 Hemolysis of peptide P2 at indicated concentration**

**
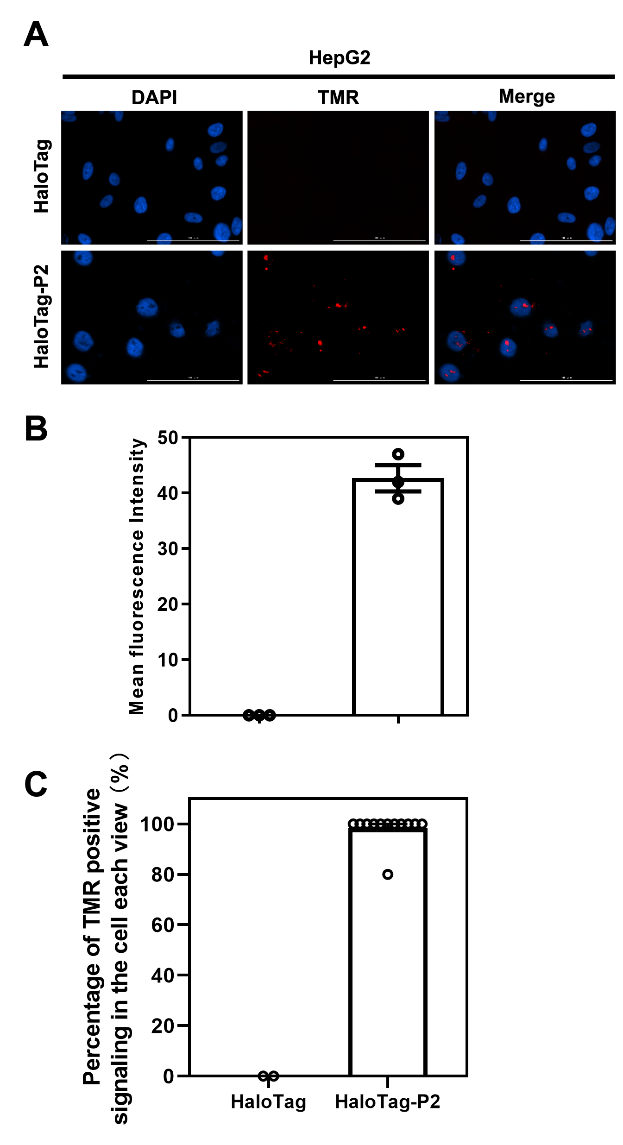
**

**Figure S8 Peptide P2 mediated HaloTag delivery**

1. Fluorescence images of 0.4 µg/ml of HaloTag-P2 labelled with TMR substrate (0.25 µM).
2. Fluorescence intensity of 0.4 µg/ml of HaloTag-P2 labelled with TMR substrate (0.25 µM).
3. Percentage of TMR positive cells treated with proteins.
